# Some Points in the Diagnosis and Treatment of Pneumonia

**Published:** 1904-12

**Authors:** John R. Stivers

**Affiliations:** Visiting Physician to the Kings County Hospital


### SELECTIONS AND ABSTRACTS.

### Some Points in the Diagnosis and Treatment of Pneumonia.

By John R. Stivers, M.D.,
Visiting Physician to the Kings County Hospital.

In a brief paper it is impossible to treat exhaustively a subject as broad as pneumonia, so it is my purpose to lay before you a few facts pertaining to this disease in such a manner as to invite friendly criticism and discussion.

According to recent teachings, pneumonia may be defined as an acute infectious disease, affecting the small bronchial tubes and air vesicles of the lungs, together with a constitutional poisoning resulting from the absorption of toxins produced by the germs. The germ pneumococcus was discovered by Sternberg, who first found it in his own sputum. While this germ is frequently found in the sputum of persons in health, it is always present in pneumonic sputum; and it is this fact that justifies us in regarding pneumonia as an infectious disease.

Just a word in regard to the pneumococcus. Experiment has shown that the germ retains its virulence for a considerable time in the dried state, and that such germs have produced the disease in rabbits by inoculation. I maintain, therefore, that the sputum from pneumonic patients should be disinfected or destroyed just as carefully as that from tubercular patients. Numerous cases come to our notice where persons have been attacked after apparently having been exposed to the disease.

Men are more frequently attacked than women, probably because of their greater tendency to intemperate habits, and the more exhausting and exposed occupations to which they are subjected.

Pneumonia frequently complicates other acute diseases, such as typhoid fever, whooping-cough, measles, and in fact all the exanthematous diseases, and is frequently the primary cause of death when it complicates any of those conditions. One attack of pneumonia does not protect against another, but rather predisposes toward future outbreaks.

In regard to the pathology, it is only necessary to say that the different stages of the disease are characterized by congestion, red hepatization, gray hepatization and resolution. All these conditions may exist at the same time in different portions of the lung.

While we have all seen many cases of pneumonia, it being a disease common to all seasons of the year, and affecting people in all conditions of life, both old and young, rich and poor, still the diagnosis in many cases is a matter which baffles the skill of the most expert diagnostician. In our student days we became familiar with the history, symptoms and physical signs of the disease, and the day we graduated we felt that the management of so common a disease as pneumonia would be among the easiest of our duties. The word-picture of a classical case, beginning with a chill, succeeded by high fever, pain under the nipple, a dry cough, with rapid respiration, the crepitant råle and rusty sputum, followed very soon by dullness on percussion, and bronchial breathing, left no room for doubt in our minds as to the ease with which those cases could be determined. However, the irregularity of the history and symptoms often render it necessary to be most skilled in the use of the physical signs, palpation, percussion and auscultation, to determine the presence or absence of pneumonic condition.

The symptoms alone are not always an accurate index to the pathological conditions existing in the lung. To demonstrate this fact, I will report briefly one or two cases that have come under my observation. The first case was that of a man, aged forty, of intemperate habits, who came into my office complaining that "he did not feel well." His temperature was 102 degrees Fah., pulse 120, and breathing labored but rapid. Examination of the chest showed that he had a pneumonia involving the whole of the right lung. He said that he had been sick for about four days, but had not stayed in the house; in fact, the day before coming to my office he had been in court, doing jury duty.

I advised him to go immediately to the hospital, where he died about twenty-four hours later. The post-mortem examination showed complete consolidation of the right lung with consolidation beginning in portions of the left lung.

Another case was that of a man twenty-six years of age, who came into my service at the Kings County Hospital a little more than a year ago. He stated that he had been sick for about five days, and that he had been unable to obtain admission to one of the hospitals down town, where he had applied, and that he had walked from there to the Kings County Hospital, a distance of fully three miles.

His temperature when first seen was 104 degrees Fah., pulse 130, respiration labored and the cough troublesome. Physical examination showed that he had complete consolidation of the right lung. The patient was put in bed and given the treatment which seemed to be indicated by the conditions. He made a good recovery in due time.

Many more cases of a similar nature might be recited, but these two serve to illustrate the disproportion between the symptoms and the actual pathological conditions.

In children there are two sources of error in diagnosis. The disease may be entirely masked by the cerebral symptoms and the case mistaken for one of meningitis. In fact, in those cases, there may be but few indications of pulmonary trouble.

The other condition is pleurisy with effusion, in which the physical signs in children are often misleading. In the latter case the exploratory needle will decide the question.

In making our diagnosis, it is unsafe to rely upon the symptoms alone, but we must weigh carefully the evidence produced by an intelligent examination of the chest. Percussion and auscultation are of greatest importance. Any deviation from the normal percussion note points to an increased density of the lung. Such areas should be carefully explored, noting the extent of dullness, and whether the dullness is absolute or only relative and whether it is produced by deep or superficial percussion.

Having determined by percussion that there is an area of dullness, and the extent of it, auscultation either with or without the aid of a stethoscope, will help us to find out the stage to which the disease has progressed. If the breathing be pure bronchial, we know that the lung must be consolidated. If it be broncho-vesicular, we know that either the lung is not completely consolidated, or that a central portion is consolidated, but covered by normal lung. It is common to find all varieties of breathing within a small area, for in a broncho-pneumonia, the so-called catarrhal pneumonia, the consolidated portion may be very small, perhaps not larger than an egg. This is particularly the case in children.

In regard to treatment, the fact that so many different lines have been recommended by different writers proves that no one system is universally adopted. We are compelled to admit that pneumonia is a self-limited disease, which can neither be aborted nor cut short by any known means at our command.

An observer once stated that a majority of cases of pneumonia would recover with good treatment, without any treatment, or in spite of bad treatment. Notwithstanding that statement, I believe the treatment to be of great importance, and by treatment I mean not only the administration of drugs, but the general management of the case. There is, no doubt, a tendency on the part of many practitioners to overtreat or overdose their patients. It is better to err on the side of giving too little rather than too much medicine. It is here that experience, skill and good judgment aid in discriminating in the use of drugs.

In the treatment of an ordinary case of pneumonia it is my custom, first, to sweep out the alimentary canal with a dose of calomel, either ten grains of the powder, or a much smaller dose if the triturates be used. This should always be followed by saline. In the early or congestive stage one-half drop of the tincture of aconite given every hour, for several doses, gives good results by reducing arterial tension and I believe can always be used with benefit.

After the first day or two the aconite may be discontinued or diminished in frequency and potassium iodide in two grain doses every three hours substituted.

When to give stimulants is a question which we must decide. Whether or not it is better to whip up an already overworked heart is a condition which confronts us. Undoubtedly many cases do well without *any* stimulation, and in fact it has been stated that many cases have resulted fatally because of being overdosed with whisky.

While there is no general rule that can be applied to all cases, it is my belief that alcoholics and persons in feeble health should always be stimulated, and that it should be begun rather early before the heart shows signs of weakening.

In other cases the physician's judgment will decide when and how many stimulants to use.

If it is decided that the patient needs stimulation, strychnine and whisky will be found most effective.

At times when there is a sudden onset of cardiac weakness one of the diffusive stimulants may be used for quick results. The aromatic spirits of ammonia I consider the most valuable.

During the stage of resolution potassium iodide and muriate of ammonia, three grains of each, every four hours, will help to liquefy the expectoration. Water to drink should be given freely.

If the temperature be high cold sponging may be used to reduce it, but never the coal-tar products. Oxygen, I believe, is a remedy of great value, if used at the proper time and in the proper manner. Let us see how the administration of oxygen may benefit the patient. The function of the lung is to supply oxygen to the blood. The oxygen is given up in exchange for carbon-dioxide. If one lung be incapacitated the remaining lung can not supply oxygen in sufficient quantity to completely oxygenate the blood, and thereby results an excess of carbon-dioxide. Carbon-dioxide stimulates the respiratory center in the medulla and the patient suffers from shortness of breath and cyanosis.

Now, if oxygen be administered it relieves the excess of carbon-dioxide, and consequently enables the patient to breathe more freely, and restores the equilibrium of the circulation. Allowing the patient to inhale one minute in every five or ten will usually be sufficient. The fact that many times when oxygen has been used in severe cases it has been withheld until there was absolutely no hope has cast a doubt in the minds of many as to the value of the remedy.

An old method of treatment of the early or congestive stage, which still has many advocates, is the application of dry or wet cups. It is certain that the withdrawal of a small quantity of blood from the surface over the affected side relieves pain and permits of more freedom of respiration.

In regard to the use of opium in pneumonia there are many arguments, both for and against it. In the beginning of the attack a few doses of opium in some form may be given safely and with benefit to the patient to relieve the shock consequent upon the sudden onset of the disease. But as opium tends to a congestion of the lung and locks up the secretions both from the lung and the intestinal tract, I believe the drug should be withheld in the latter stages, except in those cases where there is considerable involvement of the pleura and opium is demanded for its anodyne effect. In central pneumonia the patients suffer but little pain and no anodyne is required.

Theoretically the external application of ice ought to be a remedy *par excellence*, for not only does cold prevent inflammation, but, further, the germs of pneumonia will not grow in a low temperature. My own experience with the use of ice as a therapeutic agent in this disease has been limited to a few cases, but the general results have not been satisfactory.

In the first place, it is a difficult matter to adjust and keep in position an ice-bag just where it is most needed, and besides, the patient usually finds it exceedingly disagreeable. However, in hyperpyrexia, cold sponging is a most efficient agent.

Carbonate of creosote is one of the newer remedies recommended for pneumonia, for which much has been claimed by some who have used it. It is recommended to give the drug in ten to fifteen minim doses every two or three hours. It may be put in capsule or given in mucilage acacia, mixing each dose as needed.

Some have stated that by the prompt use of this drug attacks of pneumonia have been aborted. Others claim that the duration of the attack has been shortened and that the fever ran a mild course.

I used the drug last year in a few cases, but my experience failed to bear out the extravagant claims made for it by its enthusiastic advocates. It is difficult to determine the curative power of any drug in this disease, but it is probable that by its antiseptic property, carbonate of creosote has some effect on the general toxemia, and that it also reduces the temperature. I hope to give it further trial.

An agent that I believe well worth mentioning, but which I shall very briefly refer to, is the saline infusion. They certainly do much good both in eliminating the poison and in helping to tide over a critical period of cardiac depression. As much as one or two pints may be injected, the thigh being the favorite seat of injection.

The diet of a pneumonia patient should receive careful attention, and should be nutritious, but in a form easily digested. It should consist of milk, broths, albumen water and coffee, given in small quantities, but at frequent intervals. It is well to encourage the patient to drink freely of water, either plain or carbonated. A pleasant drink may be made by adding one dram of citric acid to two ounces of simple syrup, and of this, putting two teaspoonfuls into a glass of water, allowing the patient to drink *ad libitum.*

I have outlined in a general way my plan of treatment of pneumonia. But after reading a number of standard text-books, and referring to medical literature in general, and after talking with a large number of active practitioners, I am forced to conclude that we have no line of treatment that will successfully combat the disease in many cases. We may control the fever and we may support the heart, but as yet we have no known agent that will limit or control the toxemia.

We must, therefore, treat the individual and not the disease.

Pneumonia is declared to be the most fatal of all acute diseases, and the mortality rate has not appreciably changed in the last ten, twenty or thirty years. Hospital statistics give the mortality from twenty to forty per cent.

The death-rate in hospitals is larger than in private practice, because, as is well known, alcoholics furnish a large percentage of the pneumonia cases in hospitals, and in that class of patients the disease is extremely fatal.

The death-rate in private practice is probably from ten to twenty per cent. Besides alcoholics, the disease is very fatal in the old and in those whose habits and previous conditions in life have lowered their vitality.

Not being able to cure the disease, we must look to preventive medicine and possibly to some protective serum to enable us to make a more favorable showing as regards the mortality.—*Brooklyn Medical Journal.*

